# Aliphatic Anion Exchange Ionomers with Long Spacers and No Ether Links by Ziegler–Natta Polymerization: Properties and Alkaline Stability

**DOI:** 10.3390/molecules27020395

**Published:** 2022-01-08

**Authors:** Raul Andres Becerra-Arciniegas, Riccardo Narducci, Gianfranco Ercolani, Luca Pasquini, Philippe Knauth, Maria Luisa Di Vona

**Affiliations:** 1 Department of Industrial Engineering and International Laboratory “Ionomer Materials for Energy”, University of Rome Tor Vergata, Via del Politecnico 1, 00133 Roma, Italy; divona@uniroma2.it; 2 Aix-Marseille Univ, CNRS, MADIREL (UMR 7246) and International Laboratory “Ionomer Materials for Energy”, Campus St Jérôme, 13013 Marseille, France; luca.pasquini@univ-amu.fr (L.P.); philippe.knauth@univ-amu.fr (P.K.); 3 Department of Chemical Sciences and Technologies, University of Rome Tor Vergata, Via della Ricerca Scientifica, 00133 Roma, Italy; ercolani@uniroma2.it

**Keywords:** poly(vinylbenzylchloride-co-hexene), PVA blend membranes, alkaline stability, copolymer, ionic conductivity

## Abstract

In this work we report the synthesis of poly(vinylbenzylchloride-co-hexene) copolymer grafted with N,N-dimethylhexylammonium groups to study the effect of an aliphatic backbone without ether linkage on the ionomer properties. The copolymerization was achieved by the Ziegler–Natta method, employing the complex ZrCl_4_ (THF)_2_ as a catalyst. A certain degree of crosslinking with N,N,N′,N′-tetramethylethylenediamine (TEMED) was introduced with the aim of avoiding excessive swelling in water. The resulting anion exchange polymers were characterized by ^1^H-NMR, FTIR, TGA, and ion exchange capacity (IEC) measurements. The ionomers showed good alkaline stability; after 72 h of treatment in 2 M KOH at 80 °C the remaining IEC of 76% confirms that ionomers without ether bonds are less sensitive to a S_N_2 attack and suggests the possibility of their use as a binder in a fuel cell electrode formulation. The ionomers were also blended with polyvinyl alcohol (PVA) and crosslinked with glutaraldehyde. The water uptake of the blend membranes was around 110% at 25 °C. The ionic conductivity at 25 °C in the OH^−^ form was 29.5 mS/cm.

## 1. Introduction

Anion exchange membranes (AEMs) are ion conducting materials used in several applications like anion exchange membrane fuel cells (AEMFCs) [1,2,3,4], water electrolysers [5], water treatments, and redox flow batteries [6,7]. AEMFCs are promising to decrease the cost of fuel cell devices because less expensive and more abundant electrocatalytic materials, such as nickel, iron, silver, or carbon nanotubes, can catalyze the oxygen reduction reaction (ORR) in basic conditions [8].

In redox flow batteries, anion exchange membranes attain low ion permeability and high selectivity, which are critical conditions for the success of the device [9]. However, the insufficient stability of currently existing hydroxide conducting ionomers in alkaline media is an important challenge for this field [3,10]. Regarding the stability of cationic groups, ammonium presents several degradation mechanisms; the second order nucleophilic substitution S_N_2 is difficult to prevent and depends on the strength and concentration of the nucleophile and ammonium moiety [11]. The Hoffman elimination (E2) can be avoided using ammonium groups without β-hydrogens or structures where it is impossible to reach an anti-periplanar conformation; the ylide formation is difficult to inhibit [12,13,14]. The backbone is responsible for the mechanical properties and among many other commercial polymers, poly(2,6-dimethyl-1,4-phenylene oxide) (PPO) is stable in alkaline conditions and is easy to functionalize [15,16,17]. However, the functionalization can alter the charge distribution and destabilize the ether linkage which becomes sensitive to the attack of OH^−^; consequently, the conductivity is lowered and the polymer becomes fragile [11]. Various strategies have been explored to decrease the degradation [18] including a change of the polymer backbone [19,20], the introduction of long side chains, [21,22,23,24], and the delocalization of the positive charge [13,25]. Similarly, the use of composites with stable inorganic materials in alkaline environments such as hydrotalcites have shown an improvement in mechanical properties [26,27,28,29].

Recently, important advances have been made on AEMs based on aromatic polymers without ether bonds. For instance, Lee et al. [30] introduced a new synthetic design to obtain bromoalkyl-tethered poly(biphenyl alkylene)s by acid-catalyzed Friedel−Crafts polycondensation. AEMs were obtained with good chemical stability in alkaline media. In 2018 Pham et al. [31] improved thermal stability and hydroxide ion conductivity with the introduction of N-spirocyclic quaternary ammonium cations; the thermal stability was attributed to the high aromaticity and rigidity of the cationic copolymers. Other types of AEMs with similar polymeric matrix and architectures have been explored obtaining exceptional chemical stability and hydroxide ion conductivity [32,33,34].

In this study we focus on the backbone, in particular on the study of how the absence of ether bonds in the polymer matrix improves the chemical stability of anion exchange ionomers, synthesizing the copolymer poly(VBCl-co-hexene). We chose the Ziegler–Natta polymerization (ZNp) because this technique of polymerization is the most suitable for the synthesis of poly alpha-olefins [35] and co-polymerization of alpha-olefins with styrenic monomers [36]. It has also been employed for the homopolymerization of styrenic monomers [37]. The ZNp allows the control of the stereospecificity, depending on the nature of the employed catalyst [38,39]. This chemical reaction is one of the most important at an industrial level for the synthesis of plastic materials and derivatives, such as high-density polyethylene (HDPE), linear low-density polyethylene (LLDPE), syndiotactic and isotactic polypropylene [40], among others. An eventual production of ionomers though this synthetic method would be inexpensive, because the ZNp has already been developed at large-scale. Zhu et al. [41] in 2019 reported for the first time the use of classical ZNp for the synthesis of AEMs. They employed TiCl_4_ activated with methylaluminoxane (MAO) as a catalyst. The authors found a remarkable stability with just 15% to 20% of degradation after 1000 h in 1M NaOH at 80 °C. In 2019, a great advance in AEMs was achieved with the use of poly(norbornene), a polymer with an aliphatic backbone synthesized by different polymerization techniques such as Pd(II)-catalyzed addition polymerization [42] or ring-opening metathesis polymerization [43]. Membranes based on poly(norbornene) were highly resistant to the degradation. This progress make the use of aliphatic backbones in AEMs attractive.

In this work, we employ the ZrCl_4_ (THF)_2_ complex as a catalyst. This complex was tested for the first time in 2003 by Proto et al. [44], for the propylene, ethylene, and styrene homopolymerizations. They obtained high molecular weights for ethylene and propylene, as well as a high degree of iso- and syndio- tacticity. The good catalytic performance of this complex was associated with the presence of neutral ligands (electron donors) such as THF or diethyl ether.

We aimed in this work: (i) to synthesize the new copolymer poly(VBCl-co-hexene) with no ether linkage looking for an increased stability in strong alkaline conditions, (ii) to obtain a high ionic conductivity because of the introduction of long spacer chains into the backbone and with the introduction of ammonium groups on long side chains, (iii) to obtain a blend membrane of poly(VBCl-co-hexene) and polyvinyl alcohol (PVA), presenting a plastic behavior similar to Nafion^®^ due to the use of a flexible aliphatic backbone in both polymers. 

## 2. Results and Discussion

### 2.1. Synthesis and Characterization of the Copolymer Poly(VBCl-co-hexene)

**Figure 1:**
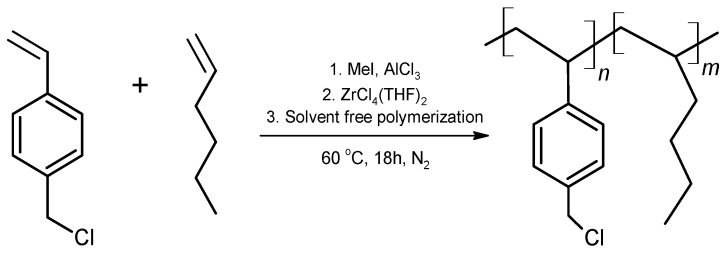
Ziegler–Natta polymerization for the synthesis of poly(VBCl-co-hexene).

The reaction carried out for the synthesis of the copolymer poly(VBCl-co-hexene) is shown in Figure 1.

**Figure 2:**
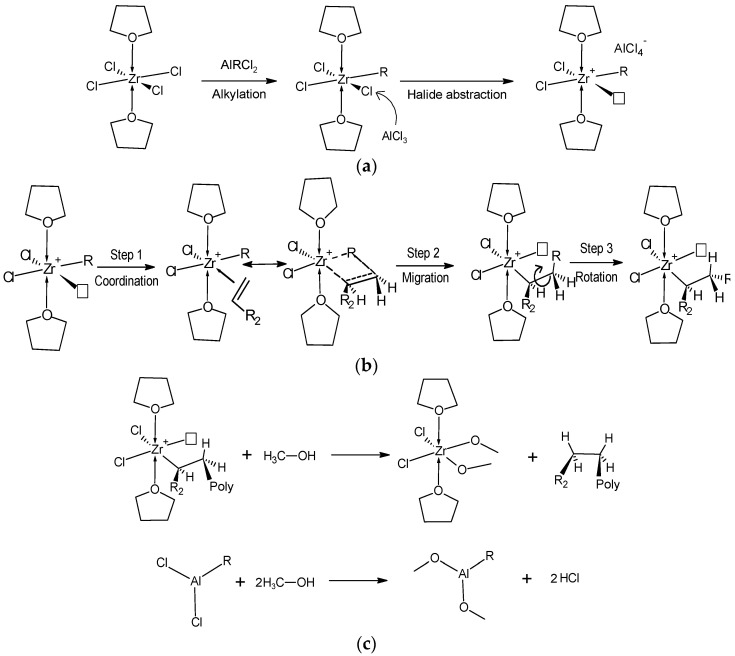
Ziegler–Natta polymerization: (**a**) Activation of the catalyst. The arrows represent the coordination bonds and the square, a vacant position; (**b**) Propagation reaction. R_2_ represents the pendent group of the monomer, it can be aliphatic or aromatic; (**c**) Termination reaction.

One of the most accepted mechanisms for the Ziegler–Natta polymerization reaction was established by Cossee in 1964. The activation of the catalyst is reported in Figure 2a [45].

When the monomers enter into contact with the activated catalyst, mainly three processes take place (Figure 2b). (i) The first step is the coordination of the monomer to the transition metal. (ii) The second step involves the “migratory insertion” of the alkyl group (R) from the Zr atom to a carbon atom of the olefin. This process takes place by a concerted reaction; at the same time new active sites are formed. (iii) The last step is the rotation about the C_α_-C_β_ single bond of the extended alkyl group. This movement of R allows another monomer to coordinate to zirconium.

The growth of the polymer chain (propagation reaction) is carried out by repeating the three steps until a termination reaction takes place (organometallic catalytic cycle). The termination reactions can occur through different processes such as: (a) β-elimination from the polymer chain, forming metal hydride; (b) β-elimination with hydrogen transfer to the monomer; (c) hydrogenation. In order to deactivate the catalyst, we employed a solution of 2% of HCl in methanol. The possible products obtained are shown in Figure 2c. The alcohol may react with a Ziegler–Natta cocatalyst such as AlR(Cl)_2_ to produce aluminum alkoxy/hydroxy compounds and an alkane. The aluminum alkoxy/hydroxy compounds cannot function as an effective cocatalyst and thus the polymerization catalyst is deactivated [46].

### 2.2. ^1^H-NMR Analysis

**Figure 3:**
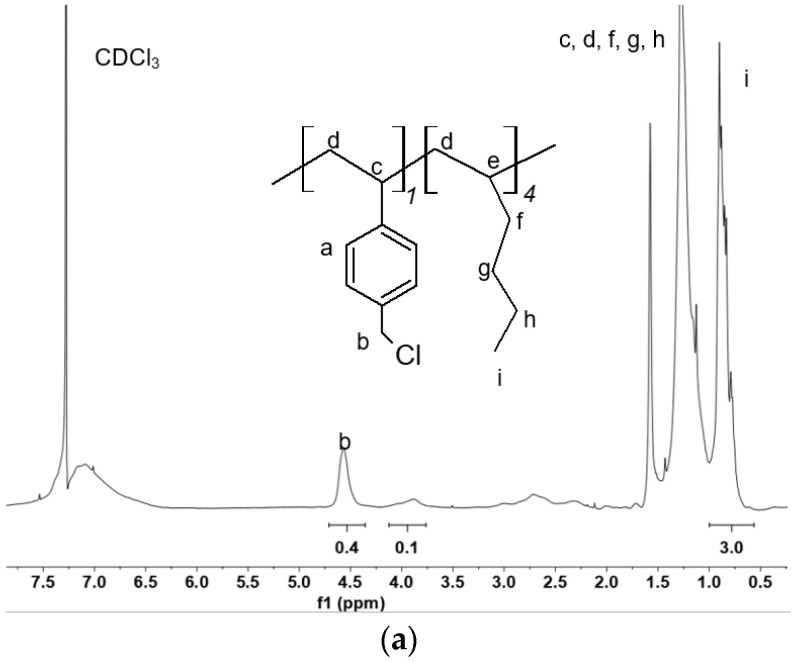

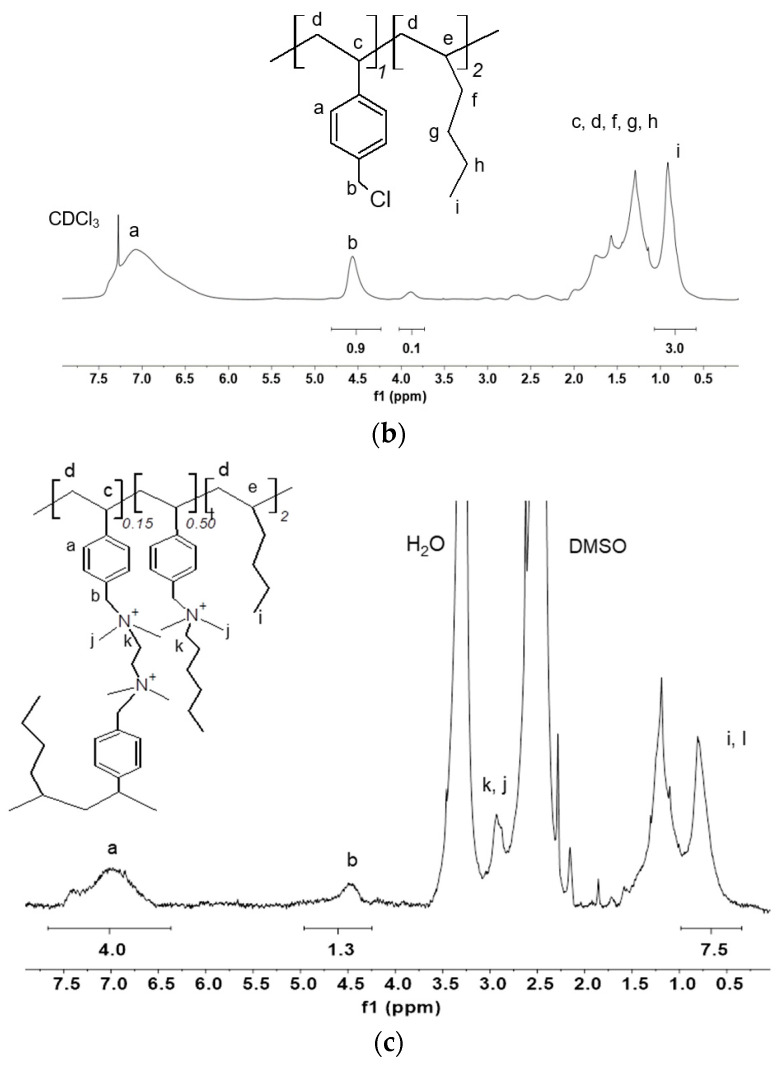
^1^H-NMR spectra of: (**a**) Copolymer poly(VBCl-co-hexene) synthesized with a ratio hexene:VBCl 4:1 in CDCl_3_; (**b**) Copolymer poly(VBCl-co-hexene) synthesized with a ratio hexene:VBCl 2:1 in CDCl_3_; (**c**) Aminated and crosslinked ionomer in d_6_-DMSO.

Copolymers were synthesized employing different molar ratios of monomers hexene:VBCl 4:1 and 2:1. The ^1^H-NMR spectra of the samples are shown in Figure 3a,b. Figure 3a corresponds to the copolymer synthesized with a monomer ratio 4:1. The signal (a) between 6.5 and 7.3 ppm is attributed to aromatic hydrogens (4.0, 4H) of the VBCl portion. The signal (i) around 0.9 ppm (3H) corresponds to the methyl group coming from the hexene monomer; we attributed to it an integral of 3. The peak (b) at 4.6 ppm is related to the chloromethylated moieties (0.4, 2H), while the signal around 3.8 ppm (0.1, 2H) is characteristic of the Ph-CH_2_-Ph portion due to a side Friedel–Crafts reaction between the chloromethylated moieties and aromatic groups [47]. The integral of aromatic hydrogens present in VBCl is generally used to estimate the ratio between the monomers [48], in our case, the aromatic hydrogens overlap with the signal of the solvent (CDCl_3_) which makes an accurate assessment impossible. Considering the initial ratio of hexene and VBCl (4:1), 3H (i) of the CH_3_ group correspond to 0.5H of benzylic moieties (b + PhCH_2_Ph), in accordance with the spectrum reported in Figure 3a. Therefore, n = 1 and m = 4. The theoretical degree of functionalization (DF) should be 0.20, due to the presence of the Friedel–Crafts reaction the effective DF is 0.16.

The Figure 3b shows the ^1^H-NMR spectrum of the sample synthesized with a ratio hexene:VBCl 2:1. The spectrum shows similar signals to the product analysed above. The ratio of hexene:VBCl in the copolymer calculated with the ^1^H-NMR spectrum considering both the signal at 4.6 and 3.8 ppm, is exactly 2:1, therefore, n = 1 and m = 2. The theoretical DF is 0.33, the experimental one is 0.3.

### 2.3. Catalytic Activity of ZNp

The polymerization activity, reported in Table 1, is lower in comparison with catalytic systems described in the literature for different homopolymers such as poly(propylene) and poly(styrene) prepared with the same catalyst [44,49,50]. We attribute the different catalytic activity to the absence of solvent in the system (solvent free reaction); as the reaction progressed, the viscosity increased, limiting the monomer transport to the active centers of the catalyst. Furthermore, we employed a mixture of AlCl_3_ + MeI as cocatalyst, which is probably less efficient than the MAO used in the reported works. FTIR analysis (see later) showed the presence of isotactic poly(1-hexene) segments, demonstrating the stereospecificity of ZrCl_4_(THF)_2_ for the ZNp, as was previously observed for the synthesis of aliphatic homopolymers. The good stereospecificity of the catalyst was attributed by Proto et al. to the presence of neutral Lewis bases bonded to the metal [44].

The numbers of branches per 1000 C are shown in Table 1; they were calculated assuming that only the Friedel–Crafts reaction took place as the source of branching. This assumption is in good agreement with the results reported by Proto et al., which found that the ZrCl_4_(THF)_2_ ZNp catalyst generates mainly linear polymers [44,50]. 

**Figure 4:**
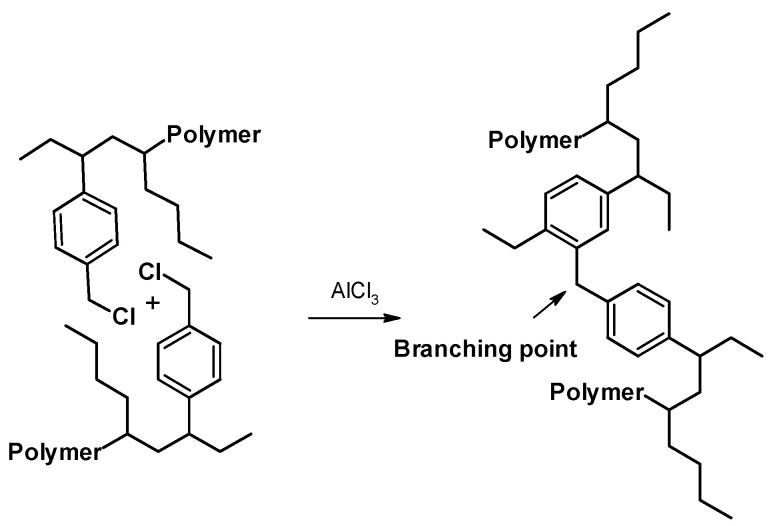
Illustration of the possible branching reaction.

The branching reaction between the polymeric chains by Friedel–Crafts is due to the presence of AlCl_3_ in the system as reported in Figure 4. The appearance of a signal at 3.9 ppm (Figure 3a,b) confirms the presence of a crosslinked structure.

The Friedel–Crafts reaction can be avoided by using different cocatalysts such as MAO. The degree of branching might be controlled by the amount of VBCl monomer, as observed in Table 1. The copolymers reported in this manuscript present quite a low degree of ramification in comparison with different polymers reported in the literature [51,52]. 

### 2.4. Synthesis of the Ionomer Based on the Copolymer Poly(VBCl-co-hexene)

**Figure 5:**
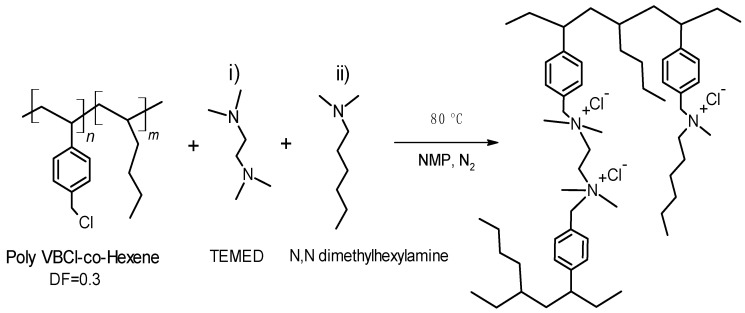
Pathways to synthesize the crosslinked ionomer based on poly(VBCl-co-hexene).

For the synthesis of the ionomer, we chose the copolymer with DF = 0.30, initially exploring the quaternization with TMA and N,N-dimethylhexylamine. Unfortunately, the membrane made with TMA became soluble in water, while the one with N,N-dimethylhexylamine suffered of excessive swelling deteriorating its mechanical properties. These results are in agreement with literature reports that ionomers with aliphatic backbones present excessive swelling attributed to a low mass of monomers and a low interaction between the polymer chains. To avoid this issue, two main approaches were reported in the literature: (1) the decrease of the IEC below 1.6 meq/g [18,19,53,54] and (2) the formation of crosslinked polymers [42,48,55,56,57,58]. We chose to crosslink the copolymer (DF = 0.3) with a diamine in order to increase the IEC and at the same time avoid excessive swelling. The ionomer was synthesized in two steps (Figure 5): (i) copolymer crosslinking with TEMED in defect; (ii) quaternization of unreacted Ph-CH_2_-Cl with N,N-dimethylhexylamine.

### 2.5. ^1^H-NMR Analysis of the Crosslinked Ionomer

The ^1^H-NMR spectrum of the crosslinked ionomer is shown in Figure 3c. The aromatic hydrogens (a) were taken as a reference (four hydrogens) considering that their fraction in the polymer is n = 1 and must remain unchanged after the quaternization reaction. 

The presence of an additional CH_3_ from the N,N-dimethylhexylamine (l) increases the value of the signal at 0.9 ppm (i + l) with respect to the sample reported in Figure 3b. Considering that n = 1 and m = 2, the peak (i) accounts for six hydrogens, the rest of hydrogens (1.5) can be assigned to (l) corresponding to 50% of the unit m, that is the quaternized chloromethyl groups. The signal (b) integrates for hydrogens at the benzylic position of Ph-CH_2_-N^+^ (65%). Accordingly, the crosslinking degree can be evaluated at around 15%. The six hydrogens of the CH_3_ groups of the ammonium moiety (j) and the two hydrogens in the alpha position of the alkyl side chain of the amines (k) are observed around 2.9 and 2.7 ppm respectively [59]. These signals overlap slightly with DMSO. 

The DAM can be evaluated at around 0.21 taking into account the 2 units of polyhexene and the unit of VBCl.

### 2.6. Infrared Spectra Analysis

**Figure 6:**
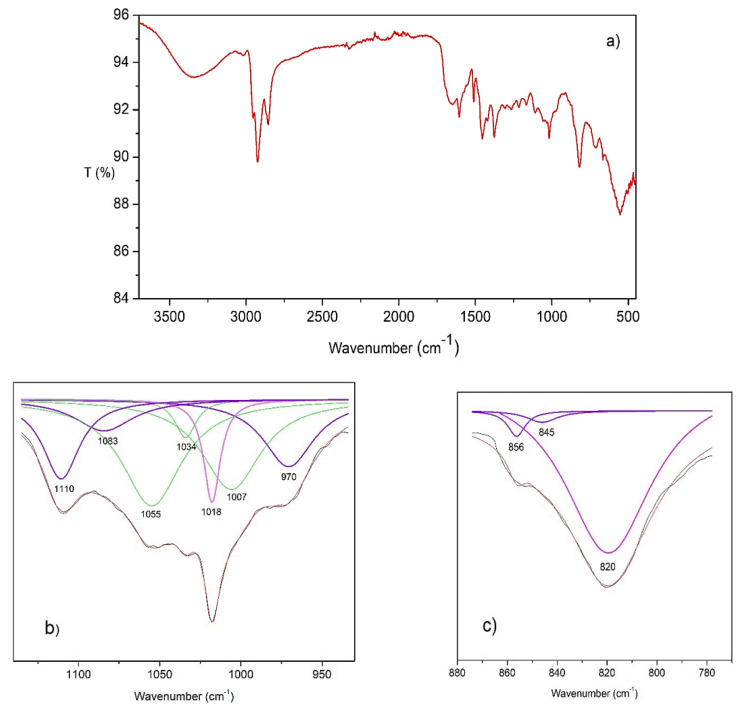
(**a**) FTIR spectrum of the crosslinked ionomer; (**b**) Zoom of the infrared spectrum between 930 and 1160 cm^−1^; (**c**) between 780 and 870 cm^−1^ and deconvolution of overlapped peaks. Signals of isotactic poly(1-hexene) segments (blue lines), signal of bending of quaternary ammonium groups (pink line), sum of peaks (red line), original signal (black line).

Figure 6a shows the spectrum of the crosslinked ionomer. The signal at 642 cm^−1^ corresponds to the stretching of C–Cl bond of benzyl chloride moieties that have not reacted during the quaternization reaction, as confirmed by the signal at 1263 cm^−1^ assigned to CH_2_Cl wagging vibrations [60]. The peaks at 1504, 1108, 1019 and 820 cm^−1^ correspond to the asymmetric C–N stretching and bending of the quaternary ammonium groups [61]. The CH_3_ groups of the R′,R-dimethyl ammonium moieties are expected to absorb in the region 3100–3020 cm^−1^; they overlap with the broad peak at 3330 cm^−1^, due to OH vibrations from water molecules in the membrane. At 1650 cm^−1^ is observed a second stretching vibration of water. There are absorption peaks at the wavenumbers of 2855 and 2924 cm^−1^ due to aromatic C–H stretching vibration absorption. At the wavenumber of 1130 cm^−1^ a shoulder is observed, corresponding to the aromatic ring in-plane deformation. There are four absorption peaks at the wavenumbers of 1605, 1456, 1419 and 1306 cm^−1^, attributed to aromatic C=C stretching vibration absorption [62,63]. The aliphatic backbone, poly(1-hexene) segments and aliphatic part of long side chain amine show two C–H stretching vibration bands around 2940 cm^−1^, overlapping with the absorption peaks of the aromatic group; there are two other stretching bands around 1456 and 1376 cm^−1^ [64]. 

It is possible to observe overlapped signals around 1018 and 820 cm^−1^ (as mentioned above they are characteristic vibrations of quaternary ammonium groups). To know the contribution of each signal, we performed a deconvolution process, the results obtained are shown in Figure 6b,c. Characteristic signals with small intensity originate from segments of isotactic poly(1-hexene); the wavenumbers at which typical vibration modes appear are: 1110, 1083, 970, 856 and 845 cm^−1^, they are shown in blue lines. The signals at 720, 1160 and 1214 cm^−1^ also correspond to isotactic poly(1-hexene). They have been observed in both homopolymers and copolymers [65,66,67]. The presence of isotacticity is in good agreement with the results reported by Proto et al. [44] for the synthesis of homopolymers employing the same catalyst used in this work.

### 2.7. Stability Test of the Crosslinked Ionomer

The degradation test of the crosslinked ionomer was made in 2 M NaOH at 80 °C. The IEC values of the ionomer (powder form) before and after degradation were obtained by acid–base titration. The remaining IEC after 72 h of aging (0.93 meq/g), amounts to 76% of the initial value (1.23 meq/g), which is higher than for ionomers based on PPO with a long spacer chain (52%) [24]. The lower degradation rate of poly(VBCl-co-hexene) corroborates that ionomers without ether groups are less sensitive to the S_N_2 attack [68]. The degradation of benzyl alkylammonium cations seems to be triggered by ether cleavage of the main chain [69,70]. Furthermore, the presence of a long side chain in the benzyl alkyl ammonium cations contributes to slowing down the S_N_2 reaction rate through steric hindrance. 

### 2.8. Thermogravimetry of the Crosslinked Ionomer

**Figure 7:**
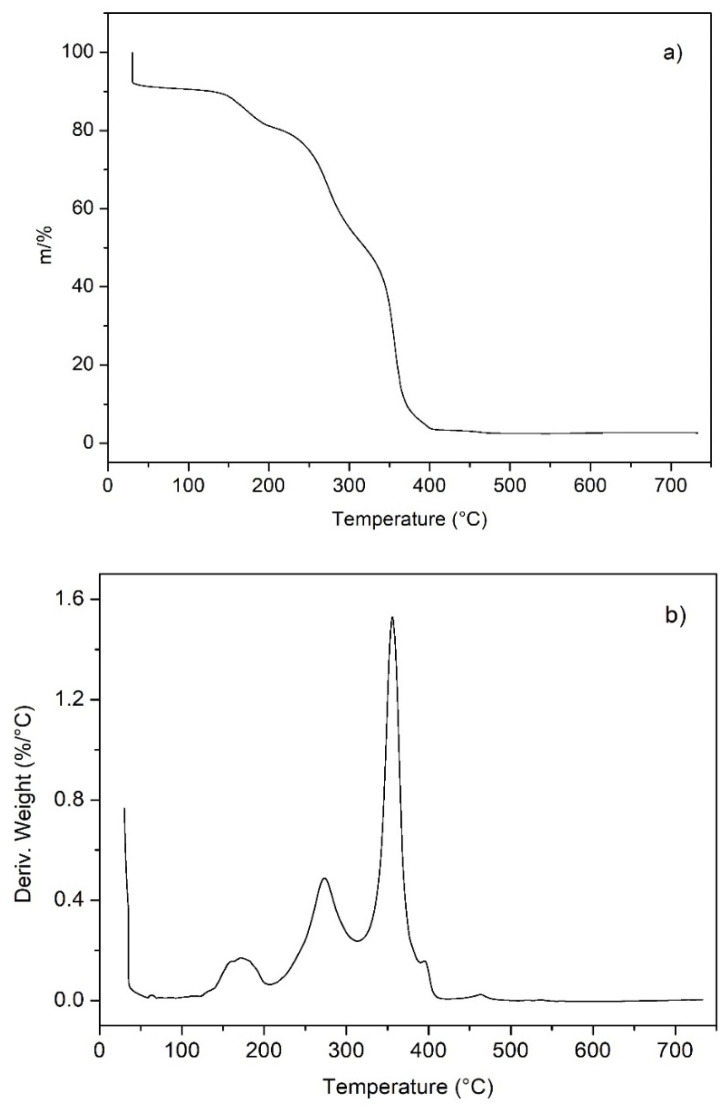
(**a**) High-resolution thermogram of the crosslinked ionomer in air; (**b**) derivative curve.

The thermogram of the crosslinked ionomer (Figure 7a) shows an initial mass decrease of about 9% of the sample mass below 100 °C, which is related to the loss of water. The large loss of water is in agreement with the high water uptake of the ionomer and the number of positive charges present on the backbone. The second broad peak between 130 °C and more than 200 °C corresponding to 12% of the sample mass can be attributed to the two amines present in the ionomer. Similar decomposition temperatures were reported in various references [71,72].

The second peak observed in the Figure 7b from 210 °C to 310 °C can be attributed to the thermal degradation of benzyl chloride moieties [73], it corresponds to about 30%. The sharp peak observed around 360 °C with a mass loss around 50% corresponds to the thermal degradation of the aliphatic poly(ethylene-co-hexene) backbone. Small peaks around 400 °C and 465 °C are probably due to the degradation of small amounts of poly(1-hexene) homopolymer formed during the polymerization reaction. These homopolymers decompose between 400 and 470 °C [74]. 

### 2.9. Water Uptake and Ionic Conductivity of Blend Membrane with PVA

The crosslinked ionomer was blended with 20% of poly(vinyl alcohol) (PVA) and was further crosslinked with glutaraldehyde in acid conditions as described in Section 3.5 to enhance its mechanical properties. The intrinsic properties of PVA, such as water solubility, nontoxicity, biodegradability, film forming properties, and low cost, make it an excellent choice. Its ability to form composites and the common use as an absorbent for alkaline solutions, giving it ionic conducting properties, make it appropriate for our purpose [75,76,77].

The WU of the blend membrane was 110% at 25 °C, while pristine PVA and cross-linked PVA presented a WU of 170% and 78% respectively. The WU is in the right order to avoid excessive swelling, but still guarantee a high hydroxide ion mobility.

**Figure 8:**
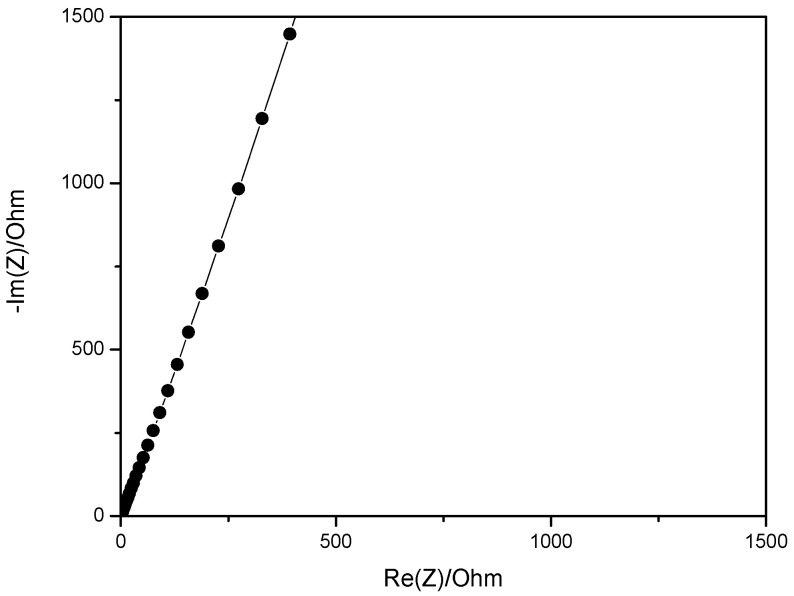
Impedance spectrum of poly(VBCl-hexene)/PVA blend membrane at 25 °C.

The impedance spectrum of the poly(VBCl-hexene)/PVA blend membrane at 25 °C is shown in Figure 8. The sample resistance is determined at the intersection of the curve with the real axis. 

The through-plane ionic conductivity of the blend membrane, calculated according to eq. 1, is reported in Table 2 as function of the temperature. 

One can notice a relatively high ionic conductivity at 25 °C showing that the ion conduction channels are well connected in the blend, because PVA presents a high hydrophilicity. The PVA blend formation is a valid strategy for obtaining high conductivity membranes that are useful for practical applications. 

The activation energy *E_A_* can be determined from the temperature dependence of the ionic conductivity *σ* according to the Arrhenius equation:

(1) $$ln\sigma =A-\frac{E_{A}}{RT}$$

The activation energy is very low (0.03 eV), which is consistent with hydroxide ion transport in aqueous solution, confirming that the PVA can participate in the ionic transport.

## 3. Materials and Methods

### 3.1. Reagents

4-Vinylbenzyl chloride (VBCl, 90%; the stabilizer was removed by washing with a 1 M NaOH solution), 1-hexene (≥99%), ZrCl_4_ (THF)_2_ (zirconium (IV) chloride tetrahydrofuran complex (1:2)), AlCl_3_ (anhydrous ReagentPlus^®^, 99%), methyl iodide (MeI, purum, ≥99.0%), poly(vinyl alcohol) (PVA, 89-98000 Mw 99% hydrolyzed), were purchased from Sigma–Aldrich. NMP, N,N-dimethylhexylamine, and N,N,N′,N′-tetramethylethylene diamine (TEMED) were purchased from TCI.

### 3.2. Synthesis of Poly(VBCl-co-hexene)

#### 3.2.1. Synthesis of the Cocatalyst

AlCl_3_ (0.23 g, 1.9 mmol) and methyl iodide (0.1 mL, 1.6 mmol) were added to a round-bottom flask and mixed under N_2_ atmosphere for 30 min.

#### 3.2.2. Catalyst Activation

The complex ZrCl_4_ (THF)_2_ (0.250 g, 0.55 mmol) and the cocatalyst were mixed. The mixture was kept under stirring conditions for 1 h at room temperature. The color changed from light yellow to dark violet after the catalyst activation.

#### 3.2.3. Random Copolymerization 

The mixture of the monomers hexene (28 mmol, 4 mL) and VBCl (14 mmol, 1.93 mL) was added to the activated catalyst with a N_2_ atmosphere. The system was kept under strong agitation at 60 °C. During the first 3 h the viscosity notably increased, the coloration of the monomer mixture changed from yellow to red. We attributed the coloration change to the formation of the complex between the catalyst and the monomer. To ensure the complete conversion, the reaction was carried out for 18 h, after this time a gel was obtained. To deactivate the complex 20 mL of 2 M HCl in 100 mL of methanol was added; the colour changed from red to strong yellow. The polymer was purified by dissolution in chloroform and precipitation in methanol. The final product was dried in an oven at 60 °C. The ^1^H-NMR spectrum in CDCl_3_ showed that the amount of chloromethylated moieties in the polymer was around 30%.

### 3.3. Crosslinking of the Copolymer with TEMED

The purified copolymer (0.7 g, 6.2 mmol) was dissolved in NMP. Then the crosslinker (TEMED 0.37 mmol, 0.06 mL) was added: the amine amount was around 20% of the chloromethylated moieties of the co-polymer. The reaction was kept at 80 °C for 26 h.

### 3.4. Ionomer Quaternization

The unreacted chloromethylated moieties were quaternized with an excess of N,N-dimethylhexylamine (0.3 mL, 1.77 mmol) for 72 h at 80 °C. After the reaction, the ionomer was precipitated in diethylether. Then the ionomer was washed with water, dried over P_2_O_5_ and the ^1^H-NMR spectrum in d_6_-DMSO was recorded.

### 3.5. Membrane Fabrication

18.75 mg of PVA (20% by weight with respect to the quaternized ionomer) was dissolved in hot bidistilled water (80 °C) under strong stirring (10 min, 600 rpm). The hot polymer solution (quaternized ionomer, 75 mg in 5 mL of NMP) was suddenly added to the PVA solution and mixed for about 10 min. After this time glutaraldehyde (5% by weight with respect to the PVA) in 50 wt% water solution was added. Later, 4 drops of concentrated HCl were added and the stirring was maintained for 10 min at RT. The solution was poured into a Teflon petri dish, and then placed in an oven at 85 °C for 3 days. The membrane was peeled off and washed with bidistilled water and then exchanged with 2 M KCl for 2 days at RT to obtain the chloride form. The membrane was washed again in bidistilled water to eliminate excess KCl. 

### 3.6. Stability Test

The stability test was performed on the ionomer in powder form. The powder was treated in 2 M NaOH at 80 °C for 72 h, then washed with deionized water to remove excess sodium hydroxide. The IEC was measured as described below.

### 3.7. Determination of Ion Exchange Capacity (IEC) by Back-Titration

The ionomers were immersed 24 h in 2 M NaOH at RT, then washed in deionized water. After drying over P_2_O_5_ for 72 h, they were weighed and immersed in a 0.018 N HCl solution. The acidic solution was then backtitrated with 0.022 N NaOH. The IEC of the pristine ionomer was 1.23 meq/g and after the degradation test the IEC decreased to 0.93 meq/g.

### 3.8. NMR Spectroscopy

^1^H-NMR spectroscopy was performed using a Bruker AVANCE 400 apparatus (400.13 MHz) using deuterated solvents (CDCl_3_, d_6_-DMSO). Chemical shifts were referenced to tetramethylsilane.

### 3.9. FTIR Spectroscopy

The FTIR spectrum was recorded in a wavenumber range 500–4000 cm^−1^ using a Perkin Elmer Spectrum 2 IR spectrometer (Waltham, MA, USA) equipped with an ATR crystal diamond module. The sample was dried before the test.

### 3.10. Thermogravimetry

The thermal stability of the polymer was investigated between 30 and 700 °C by high resolution thermogravimetric analysis using a platinum sample holder (TGA Q500, TA Instruments) with a heating rate of 3 K min^−1^.

### 3.11. Ionic Conductivity

The through-plane ionic conductivity of the blend membrane was measured between 25 and 80 °C by impedance spectrometry between 1 Hz and 6 MHz using an impedance spectrometer Biologic VSP300. The amplitude of the oscillating voltage was 20 mV. Before the test the membrane was immersed for 24 h at 25 °C in a 2 M KOH solution and rapidly washed with deionized water to remove as much as possible of excess KOH solution. The test was performed using a custom-made Swagelok cell with two stainless steel electrodes in fully humidified conditions. The ionic conductivity σ was calculated using the Equation (2):

(2) $$\sigma =\frac{th}{R*A}$$

where th and A are respectively the thickness (measured with a micrometer Mitutoyo 293-230) and the electrode area.

## 4. Conclusions

The copolymerization of VBCl and hexene by ZNp employing ZrCl_4_ (THF)_2_ catalyst was successful. The prepared copolymer and derived anion exchange ionomer were not previously reported in the literature. The stability test in alkaline conditions of the ionomer showed that the remaining IEC after 72 h was 76% of the initial value, which is higher than for the ionomers based on PPO studied previously in our laboratory, confirming that AEMs based on polymer backbones without ether linkage, such as polynorbornenes, are superior with regards to degradation in alkaline environment.

The copolymer was crosslinked with TEMED and then quaternized with N,N-dimethylhexylamine to increase the IEC and to avoid excessive swelling. Similar crosslinking approaches were reported with good results for cation conducting polymers, such as sulfonated poly(ether ether ketone).

The presence of isotactic segments of poly(1-hexene) might be employed in future works to enhance the mechanical properties of the membranes and to obtain a membrane based on just poly(VBCl-co-hexene).

The crosslinked ionomer was also blended with 20% of PVA and then crosslinked with glutaraldehyde. The handling experience showed good mechanical properties, as well as good hydrolytic stability. The conductivity is in the order of 30 mS/cm above that of PPO-based membranes reported in our previous works. The higher WU compared with PPO membranes may promote a better connectivity of hydrophilic domains facilitating hydroxide ion transport.

## Figures and Tables

**Table 1:** Catalytic activity and number of branches/1000 C obtained for the synthesis of poly(VBCl-co-hexene) at 60 °C for 18 h.

Monomer Ratio: Hexene:VBCl	Reaction Yield (%)	Polymerization Activity ^(a)^	Number of Branches/1000 C ^(b)^
4:1	43	0.021	6.1
2:1	77	0.049	4.7

^(a)^ The polymerization activity was calculated as: g polymer/(mmol Zr × h × [total monomer]). ^(b)^ Branching numbers per 1000 carbons were determined by ^1^H NMR spectroscopy.

**Table 2:** Ionic conductivity of blend membrane in OH^−^ form.

Temperature (°C)	Conductivity (mS/cm)
25	29.5
60	32.2
80	34.8
25 (After cooling down)	27.6

## Sample Availability

Samples of the compounds are not available from the authors.
